# Evolutionary dynamics of the cryptocurrency market

**DOI:** 10.1098/rsos.170623

**Published:** 2017-11-15

**Authors:** Abeer ElBahrawy, Laura Alessandretti, Anne Kandler, Romualdo Pastor-Satorras, Andrea Baronchelli

**Affiliations:** 1 Department of Mathematics—City, University of London—Northampton Square, London EC1V 0HB, UK; 2 Max Planck Institute for Evolutionary Anthropology, Department of Human Behaviour, Ecology and Culture, Leipzig, Germany; 3 Departament de Física, Universitat Politècnica de Catalunya, Campus Nord B4, 08034 Barcelona, Spain; 4 UCL Centre for Blockchain Technologies, University College London, London, UK

**Keywords:** cryptocurrency market, Bitcoin, evolutionary modelling, neutral model, complex systems

## Abstract

The cryptocurrency market surpassed the barrier of $100 billion market capitalization in June 2017, after months of steady growth. Despite its increasing relevance in the financial world, a comprehensive analysis of the whole system is still lacking, as most studies have focused exclusively on the behaviour of one (Bitcoin) or few cryptocurrencies. Here, we consider the history of the entire market and analyse the behaviour of 1469 cryptocurrencies introduced between April 2013 and May 2017. We reveal that, while new cryptocurrencies appear and disappear continuously and their market capitalization is increasing (super-)exponentially, several statistical properties of the market have been stable for years. These include the number of active cryptocurrencies, market share distribution and the turnover of cryptocurrencies. Adopting an ecological perspective, we show that the so-called neutral model of evolution is able to reproduce a number of key empirical observations, despite its simplicity and the assumption of no selective advantage of one cryptocurrency over another. Our results shed light on the properties of the cryptocurrency market and establish a first formal link between ecological modelling and the study of this growing system. We anticipate they will spark further research in this direction.

## 1. Introduction

Bitcoin is a digital asset designed to work as a medium of exchange [1,2]. Users can send and receive native tokens, the ‘bitcoins’, while collectively validating the transactions in a decentralized and transparent way. The underlying technology is based on a public ledger, or blockchain, shared between participants and a reward mechanism in terms of Bitcoins as an incentive for users to run the transaction network. It relies on cryptography to secure the transactions and to control the creation of additional units of the currency, hence the name ‘cryptocurrency’ [3,4].

After Bitcoin appeared in 2009, approximately 1500 other cryptocurrencies have been introduced, about 600 of which are actively traded today. All cryptocurrencies share the underlying blockchain technology and reward mechanism, but they typically live on isolated transaction networks. Many of them are basically clones of Bitcoin, although with different parameters such as different supplies, transaction validation times, etc. Others have emerged from more significant innovations of the underlying blockchain technology [5] (see electronic supplementary material, §S3).

Cryptocurrencies are nowadays used both as media of exchange for daily payments, the primary reason for which Bitcoin was introduced, and for speculation [6,7]. Other uses include payment rail for non-expensive cross-borders money transfer and various non-monetary uses such as time stamping [2]. The self-organization of different usages both within a single cryptocurrency and as an element of differentiation between cryptocurrencies makes the market of cryptocurrencies unique, and their price extremely volatile [8–10].

Between 2.9 and 5.8 millions of private as well as institutional users actively exchange tokens and run the various transaction networks [5]. In May 2017, the market capitalization of active cryptocurrencies surpassed $91 billion [11]. Bitcoin currently dominates the market but its leading position is challenged both by technical concerns [12–16] and by the technological improvements of other cryptocurrencies [17].

Despite the theoretical and economic interest of the cryptocurrency market [2,4,18,19], however, a comprehensive analysis of its dynamics is still lacking. Existing studies have focused either on Bitcoin, analysing, for example, the transaction network [20–24] or the behaviour and destiny of its price [9,25–30], or on a restricted group of cryptocurrencies (typically 5 or 10) of particular interest [5,17,32,31]. But even in this case, there is disagreement as to whether Bitcoin’s dominant position may be in peril [5] or its future dominance as leading cryptocurrency is out of discussion [31].

Here, we present a first complete analysis of the cryptocurrency market, considering its evolution between April 2013 and May 2017. We focus on the market shares of the different cryptocurrencies (see §4) and find that Bitcoin has been steadily losing ground to the advantage of the immediate runners-up. We then show that several statistical properties of the system have been stable for the past few years, including the number of active cryptocurrencies, the market share distribution, the stability of the ranking, and the birth and death rate of new cryptocurrencies. We adopt an ‘ecological’ perspective on the system of cryptocurrencies and note that several observed distributions are well described by the so-called ‘neutral model’ of evolution [33,34], which also captures the decrease in Bitcoin’s market share. We believe that our findings represent a first step towards a better understanding and modelling of the cryptocurrency market.

## 2. Results

### 2.1. Market description

Our analysis focuses on the market share of the different cryptocurrencies and is based on the whole history of the cryptocurrency market between 28 April 2013 and 13 May 2017. Our dataset includes 1469 cryptocurrencies, of which around 600 were active by that time (see §4).

**Figure 1.**
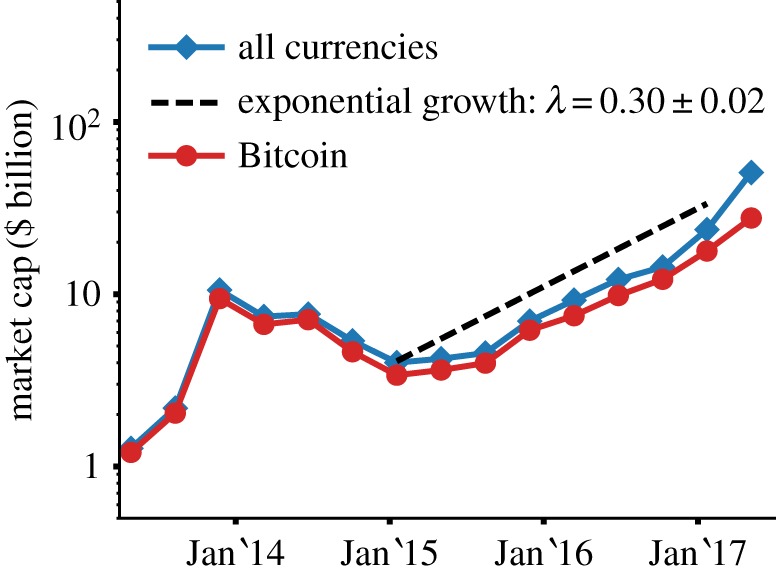
Evolution of the market capitalization. Evolution of the market capitalization over time (starting from April 2013), for all cryptocurrencies (blue line, diamonds) and for Bitcoin (red line, dots). The dashed line is an exponential curve *f*(*t*)∼*e*^*λt*^, with *λ*=0.3, shown as a guide to the eye. Data are averaged over a 15-week window.

The total market capitalization *C* of cryptocurrencies has been increasing since late 2015 after a period of relative tranquillity (figure 1). As of May 2017, the market capitalization is more than four times its value compared to May 2016 and it exhibits an exponential growth C∼exp⁡(λt) with coefficient *λ*=0.30±0.02, where *t* is measured in units of 15 weeks.

### 2.2. Decreasing Bitcoin market share

Bitcoin was introduced in 2009 and followed by a second cryptocurrency (Namecoin; see electronic supplementary material, §S1) only in 18 April 2011. This first-mover advantage makes Bitcoin the most famous and dominant cryptocurrency to date. However, recent studies analysing the market shares of Bitcoin and other cryptocurrencies reached contrasting conclusions on its current state. While Gandal and Halaburda in their 2016 study concluded that ‘Bitcoin seems to have emerged, at least in this stage, as the clear winner’ [35], the 2017 report by Hileman and Rauchs noted that ‘Bitcoin has ceded significant market cap share to other cryptocurrencies’ [5].

**Figure 2.**
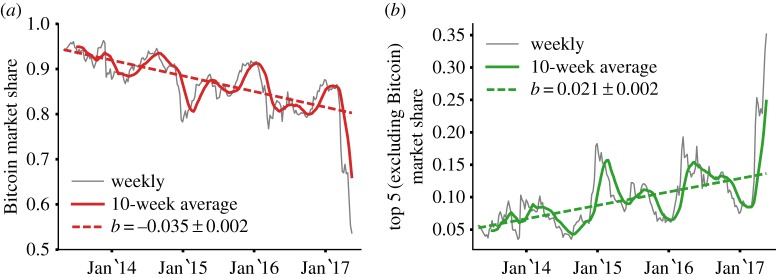
Evolution of the market share of top-ranking cryptocurrencies. (*a*) The market share of Bitcoin across time sampled weekly (grey line) and averaged over a rolling window of 10 weeks (red line). The dashed line is a linear fit with angular coefficient *b*=−0.035±0.002 (the rate of change in 1 year) and coefficient of determination *R*^2^=0.63. The Spearman correlation coefficient is *ρ*=−0.8, revealing a significant negative correlation at a significance level of 1%. (*b*) Total market share of the top 5 cryptocurrencies excluding Bitcoin sampled weekly (grey line) and averaged over a rolling window of 10 weeks (green line). The dashed line is a linear fit with angular coefficient *b*=0.021±0.002 (the rate of change in 1 year) and coefficient of determination *R*^2^=0.45. The Spearman correlation coefficient is *ρ*=0.67, revealing a significant positive correlation at a significance level of 1%.

To clarify the situation, we consider the whole evolution of the Bitcoin market share over the past 4 years. Figure 2*a* shows that Bitcoin’s market share has been steadily decreasing for the past years, beyond oscillations that might mask this trend to short-term investigations. The decrease is well described by a linear fit *f*(*t*)=*a*+*bt* with angular coefficient *b*=−0.035±0.002 representing the change in market share over *t*=1 year. Neglecting the impact of nonlinear effects and potential changes in the competition environment, the model indicates that Bitcoin’s market share can fluctuate approximately around 50% by 2025. Conversely, figure 2*b* shows that the top 5 runners-up (see electronic supplementary material, §S1) have gained significant market share and now account for more than 20% of the market.

### 2.3. Stability of the cryptocurrency market

To characterize the cryptocurrencies dynamics better, we now focus on the statistical properties of the market. We find that while the relative evolution of Bitcoin and rival cryptocurrencies is tumultuous, many statistical properties of the market are stable.

**Figure 3.**
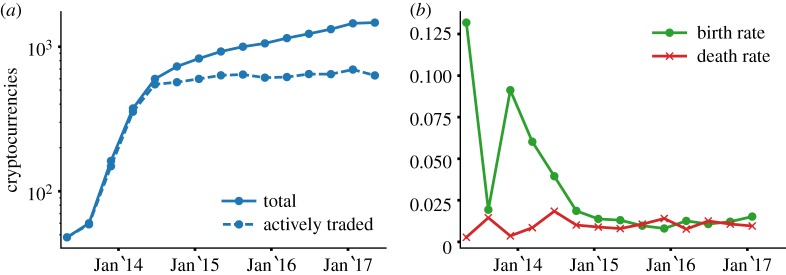
Evolution of the number of cryptocurrencies. (*a*) The number of cryptocurrencies that ever entered the market (filled line) since April 2013, and the number of actively traded cryptocurrencies (dashed line). (*b*) The birth and death rate computed across time. The birth (respectively, death) rate is measured as the fraction of cryptocurrencies entering (respectively, leaving) the market on a given week over the number of living cryptocurrencies at that point. Data are averaged over a 15-week window.

Figure 3*a* shows the evolution of the number of active cryptocurrencies across time, averaged over a 15-week window. The number of actively traded cryptocurrencies is stable due to similar birth and death rates since the end of 2014 (figure 3*b*). The average monthly birth and death rates since 2014 are 1.16% and 1.04%, respectively, corresponding to approximately seven cryptocurrencies appearing every week while the same number is abandoned.

**Figure 4.**
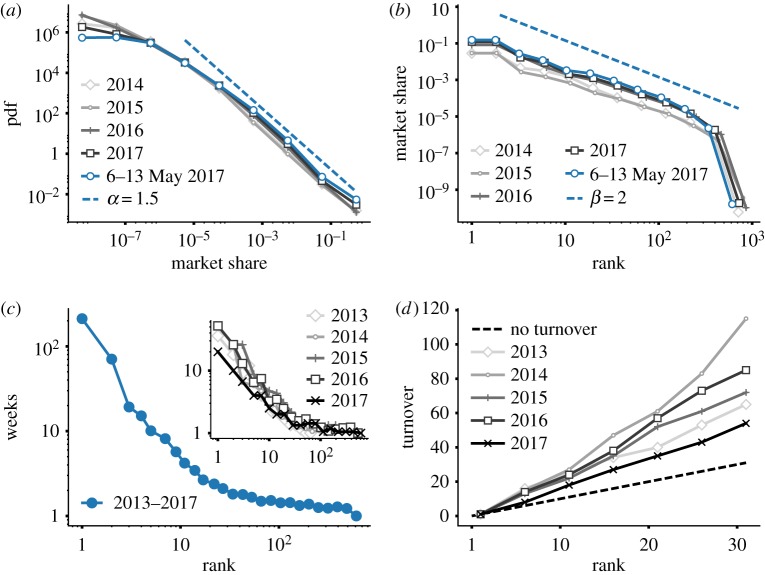
Stable properties of the cryptocurrency market. (*a*) Distribution of market share computed aggregating across a given year (grey filled lines), and over the week 6–13 May 2017 (blue thick line). The dashed line is a power law *P*(*x*)∼*x*^−*α*^ with exponent *α*=1.5. (*b*) Frequency-rank distribution of cryptocurrencies, computed aggregating across a given year (grey filled lines), and over the week 6–13 May 2017 (blue thick line). The dashed line is a power-law curve *P*(*r*)∼*r*^−*β*^ with exponent *β*=2. (*c*) Average amount of time (in weeks) a cryptocurrency occupies a given rank, computed averaging across all years (blue line), and across given years (grey lines, inset). (*d*) Turnover of the ranking distribution, defined as the total number of cryptocurrencies ever occupying a rank higher than a given rank. The measure is computed averaging across given years (grey filled lines). The 2013 and 2017 curves must be taken purely as an indication as they are computed on less than 12 months (approx. eight and four months, respectively). The dashed line has angular coefficient 1, and corresponds to the case in which the ranking of cryptocurrencies is fixed (i.e. the variable turnover captures only the initial size of the top-list).

Interestingly, the market share distribution remains stable across time. Figure 4*a* shows that curves obtained by considering different periods of time are indistinguishable. This is remarkable because the reported curves are obtained by considering data from different years as well as data aggregated on different time spans—from one week to the entire approximately 4 years of data. The obtained distribution exhibits a broad tail well described by a power-law *P*(*x*)∼*x*^−*α*^ with exponent *α*=1.58±0.12 (figure 4*a*), where the fit coefficient is computed using the method detailed in [36]. The expected relationship between the probability distribution and the frequency rank distribution predicts the latter is a power-law function *P*(*r*)∼*r*^−*β*^ with exponent *β*=1/(*α*−1) [37], yielding in our case *β*=1.72 (figure 4*b*). The empirical fit coefficient *β*=1.93±0.23 is consistent with this prediction. This was also verified for each year individually (see electronic supplementary material, §S4).

We further investigate the stability of the market by measuring the average rank occupation time (figure 4*c*), defined as the amount of time a cryptocurrency typically spends in a given rank before changing it. We find that the time spent in a top-rank position decays fast with the rank, while for low-rank positions such time approaches one week. Again, this behaviour is stable across years (figure 4*c*, inset). We also consider the turnover profile defined as the total number of cryptocurrencies ever occupying a rank higher than a given rank in period *t* (see [38] for a similar definition). Figure 4*d* shows that also this quantity is substantially stable across time.

The first rank has been always occupied and continues to be occupied by Bitcoin, while the subsequent 5 ranks (i.e. ranks 2–6) have been populated by a total of 33 cryptocurrencies with an average lifetime of 12.6 weeks. These values change rapidly when we consider the next set of ranks from 7 to 12 to reach 70 cryptocurrencies and an average lifetime of 3.6 weeks. At higher ranks, the mobility increases and cryptocurrencies continuously change position.

### 2.4. A simple model for the cryptocurrency ecology

To account for the empirical properties of the dynamics of cryptocurrencies we have discussed above we adopt the view of a ‘cryptocurrency ecology’ and consider the neutral model of evolution, a prototypical model in population genetics and ecology [33,34].

The Wright–Fisher model of neutral evolution describes a fixed-size population of *N* individuals where each individual belongs to one of *m* species. At each generation, the *N* individuals are replaced by *N* new individuals. Each new individual belongs to a species copied at random from the previous generation, with probability 1−*μ*, or to a species not previously seen, with probability *μ*, where *μ* is a mutation parameter that does not change over time [39]. Despite its simplicity, the neutral model is able to reproduce the static patterns of the competition dynamics of many systems including ecological [40] and genetics [41] systems, cultural change [42], English words usage [43] and technology patents citations [44].

In our mapping of the ecological model to the cryptocurrency market, each individual corresponds to a certain amount of dollars, while species correspond to different cryptocurrencies (see electronic supplementary material, §S2). The copying mechanism represents trading, with *μ* denoting the probability that a new cryptocurrency is introduced. Our choice of *μ* is informed by the data to yield a number of new cryptocurrencies per unit time corresponding to the empirical observation. We thus fix *μ*=7/*N*, where *N* is the population size in the model. Thus, one model generation corresponds to one week of observations, the choice of *μ* guaranteeing an average of seven new cryptocurrencies entering the system every week, as empirically observed. Finally, in contrast with most neutral models, we assume that a new species does not enter the system with a single individual but with a size proportional to the empirical average market share of a new cryptocurrency (see electronic supplementary material, §S2).

The neutral model translates in the simplest way three main assumptions [45]: (i) interactions between cryptocurrencies are equivalent on an individual per capita basis (i.e. per US dollar), (ii) the process is stochastic, and (iii) it is a sampling theory, where the new generation is the basis to build the following one. In other words, the neutral model assumes that all species/cryptocurrencies are equivalent and that all individuals/US dollars are equivalent.

**Figure 5.**
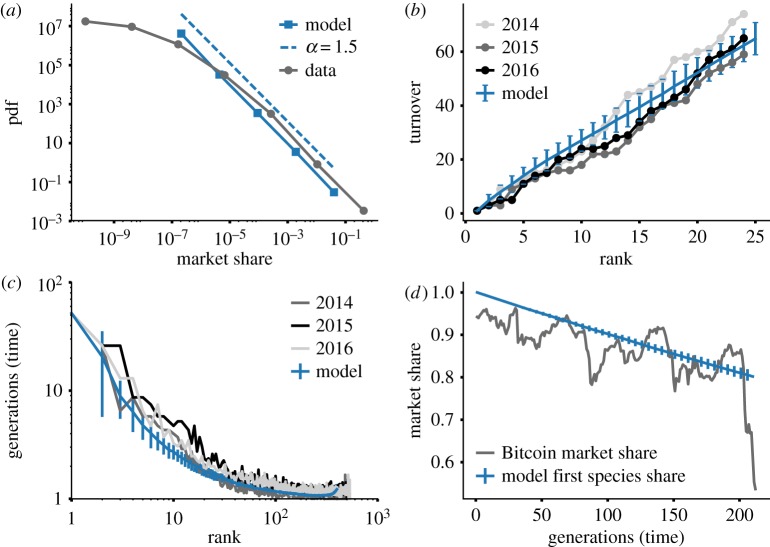
Neutral model for evolution and empirical observations. (*a*) Distribution of cryptocurrencies market shares aggregated over all years (grey line, dots) and the equilibrium distribution resulting from numerical simulations (blue line, squares) aggregated over 210 generations. The dashed line is the power-law curve *P*(*x*)∼*x*^−*α*^ predicted analytically with exponent *α*=1.5 [46]. (*b*) Turnover of the ranking distribution computed considering 52 generations of the cryptocurrencies data (grey lines, dots) and for numerical simulations (blue line). (*c*) Average number of generations of a cryptocurrency (grey lines) and a species in the neutral model (blue line) occupies a given rank. Averages are computed across 52 generations. (*d*) Evolution of the market share of Bitcoin (grey line) and the expected market share of the first species in numerical simulations (blue line). All simulations are run for *N*=10^5^ and *μ*=7/*N* starting from one species in the initial state. The size of entering species *m*, whose average *m*=15 is informed by the data, is taken at random in the interval *m*=[10,20]. Error bars are standard deviations, computed across 100 simulations. (*b*) and (*c*) Measures start at generation *g*_1_=105 (see electronic supplementary material, §S2 for variations of this parameter).

Testing the consistency between observed patterns of the cryptocurrency market and theoretical expectations of neutral theory revealed that neutrality captures well at least four features of the cryptocurrency ecology, namely:

— the exponent of the market share distribution (figure 5*a*);
— the linear behaviour of the turnover profile of the dominant cryptocurrencies (figure 5*b*);
— the average occupancy time of any given rank (figure 5*c*); and
— the linear decrease of the dominant cryptocurrency (figure 5*d*).

The neutral model generates in fact an aggregated species distribution (i.e. obtained when all generations up to the *i*th are combined together and analysed as a single population of size N*i [44,47]) that, at equilibrium, can be described by a power-law distribution *P*(*x*)∼*x*^−*α*^ with *α*=1.5 [46], in agreement with the empirical value *α*=1.58±0.12 obtained by the fitting procedure described in [36]. Figure 5*a* shows the agreement between simulations and data (same behaviour of the long tail), where simulation results are aggregated over *i*=210 generations, corresponding to 4 years of empirical observations under our choice of *μ*. The existence of a power-law phase with exponent 1.5 in the model is independent of *μ* (see electronic supplementary material, §S2, and [46]).

Furthermore, when we account for the fact that Bitcoin was originally the only cryptocurrency by setting one species in the initial state, the model captures also the remaining properties. In figure 5*b*,*c*, we compare the turnover profile and the ranking occupation times with the corresponding simulation results. We compute these quantities over a period of 52 generations, corresponding to 1 year of observations. The curve reported in figure 5*b*,*c* corresponds to measures performed between generation *g*_1_=105 and *g*_2_=156, corresponding to year 3 (2015) in the data. Crucially, however, both measures are stable in time, i.e. they do not depend on the choice of *g*_1_ (but for an initial period of high rank variability for the very first generations see electronic supplementary material, §S2). It is worth noting that the linearity of the turnover profile in figure 5*b* corresponds to a similar behaviour observed in [38] when the measure is performed between two consecutive generations. Figure 5*d* shows the observed linear decrease of the leading cryptocurrency market share, indicating that newborn cryptocurrencies mostly damage the dominating one.

## 3. Discussion and outlook

In this paper, we have investigated the whole cryptocurrency market between April 2013 and May 2017. We have shown that the total market capitalization has entered a phase of exponential growth 1 year ago, while the market share of Bitcoin has been steadily decreasing. We have identified several observables that have been stable since the beginning of our time series, including the number of active cryptocurrencies, the market share distribution and the rank turnover. By adopting an ecological perspective, we have pointed out that the neutral model of evolution captures several of the observed properties of the market.

The model is simple and does not capture the full complexity of the cryptocurrency ecology. However, the good match with at least part of the picture emerging from the data does suggest that some of the long-term properties of the cryptocurrency market can be accounted for based on simple hypotheses. In particular, as the model assumes no selective advantage of one cryptocurrency over the other, the fit with the data shows that there is no detectable population-level consensus on what is the ‘best’ currency or that different currencies are advantageous for different uses. Furthermore, the matching between the neutral model and the data implies that the observed patterns of the cryptocurrency market are compatible with a scenario where technological advancements have not been key so far (see electronic supplementary material, §S3) and where users and/or investors allocate each packet of money independently. Future work will need to consider the role of an expanding overall market capitalization and, more importantly, try to include the information about single transactions, where available, in the modelling picture.

In the immediate and mid-term future, legislative, technical and social advancements will most probably impact the cryptocurrency market seriously and our approach, together with recent results in computational social science dealing with the quantification of financial trading and bubble formation [48–51], could help make sense of the market evolution. In April 2017, for example, Japan started treating Bitcoin as a legal form of payment driving a sudden increase in the Bitcoin price in US dollars [52], while in February 2017 a change of regulation in China resulted in a $100 price drop [53]. Similarly, the exponential increase in the market capitalization (figure 1) will probably attract further speculative attention towards this market, at the same time increasing the usability of cryptocurrencies as a payment method. While the use of cryptocurrencies as speculative assets should promote diversification [31], their adoption as a payment method (i.e. the conventional use of a shared medium of payment) should promote a winner-take-all regime [54,55]. How the self-organized use of cryptocurrencies will deal with this tension is an interesting question to be addressed in future studies.

## 4. Material and methods

### 4.1. Data

Cryptocurrency data were extracted from the website Coin Market Cap [11], collecting weekly data from 157 exchange market platforms starting from 28 April 2013 up to 13 May 2017. For all living cryptocurrencies, the website provides the market capitalization, the price in US dollars and the volume of trading in the preceding 24 h. Data on trading volume were collected starting from 29 December 2013.

The website lists cryptocurrencies traded on public exchange markets that are older than 30 days and for which an API as well as a public URL showing the total mined supply are available. Information on the market capitalization of cryptocurrencies that are not traded in the 6 h preceding the weekly release of data is not included on the website. Cryptocurrencies inactive for 7 days are not included in the list released. These measures imply that some cryptocurrencies can disappear from the list to reappear later on.

### 4.2. Analysis

The following quantities characterize individual cryptocurrencies: The *circulating supply* is the number of coins available to users. The *price* is the exchange rate, determined by supply and demand dynamics. The *market capitalization* is the product of the circulating supply and the price. The *market share* is the market capitalization of a currency normalized by the total market capitalization.

Most of our analyses consider the market capitalization and market share of cryptocurrencies. These quantities neglect the destroyed or dormant coins, accounting, for example, to 51% of mined Bitcoins based on data from the period 18 July 2010 to 13 May 2012 [20].

## Supplementary Material

Supplementary materials (Electronic document)

## Data accessibility

The dataset used in this study is public and can be found in Coin Market Cap [11]. The web-scraped data can also be found on Dryad Repository as http://dx.doi.org/10.5061/dryad.38776 [56].
