# Supplementary material for: Evolutionary dynamics of the cryptocurrency market
Source: R Soc Open Sci. 2017 Nov 15;4(11):170623. doi: 10.1098/rsos.170623 (PMC5717631; doi:10.1098/rsos.170623)
Supplement: Supplementary materials (Electronic document) [file rsos170623supp1.pdf]

Evolutionary dynamics of the cryptocurrency market

Supplementary Information

Abeer ElBahrawy[1], Laura Alessandretti[1], Anne Kandler[2], Romualdo Pastor-Satorras[3], and Andrea Baronchelli[1,4*]

[1]Department of Mathematics - City, University of London - Northampton Square, London EC1V 0HB, UK
[2]Max Planck Institute for Evolutionary Anthropology, Department of Human Behaviour, Ecology and Culture, Leipzig, Germany
[3]Departament de Física, Universitat Politècnica de Catalunya, Campus Nord B4, 08034 Barcelona, Spain
[4]UCL Centre for Blockchain Technologies, University College London, UK

*To whom correspondence should be addressed, E-mail: andrea.baronchelli.1@city.ac.uk.

1 On some relevant cryptocurrencies

Table S1 provides information on some relevant cryptocurrencies, either occupying high-rank positions or early introduced in the market. Data was collected in May 2017, see below for details on the Technology column.

Table S1: **Details on the top runner cryptocurrencies in the market.** The table is generated using data collected on May 28, 2013

Name	Year	Technology	Market Cap ($)	Rank	Additional Info
**Bitcoin**	2009	Proof-of-work	35B	1	
**Ethereum**	2015	Proof-of-work	15B	2	Smart contracts
**Ripple**	2013	Distributed open source consensus ledger	8B	3	Widely adopted by companies and banks.
**NEM**	2015	Proof-of-importance	1B	4	
**Ethereum Classic**	2015	Proof-of-work	1B	5	DAO Hard-fork
**Litecoin**	2011	Proof-of-work	1B	6	
**Dash**	2014	Proof-of-work	809M	7	Gained market since early 2017. Privacy focused.
**Monero**	2014	Proof-of-work	535M	8	Gained momentum in late 2016. Privacy focused
**NameCoin**	2015	Proof-of-work	21M	58	

2 Simulations

Our choice of the mutation parameter μ is informed by the data to yield a number of new cryptocurrencies per unit time corresponding to the empirical observation. By choosing $\mu = \frac{7}{N}$, where N is the population size in the model it holds that 1 model generation corresponds to 1 week of observation (since on average 7 new cryptocurrencies enter the system every week, see main text). In Fig. S1 we show that the distribution of species sizes (see Fig. 5A in main text) has a very similar shape for a broad range of choices of μ [1].

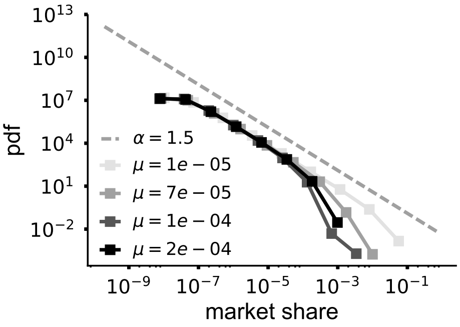

Figure S1: **Distribution of species sizes for different values of μ.** Distribution of the species sizes resulted form numerical simulations given different values of μ.

All simulations are run starting with one species in order to capture the initial dominance of Bitcoin in the cryptocurrency market. This reflects the initial state of the cryptocurrencies market, when Bitcoin was the only existing cryptocurrency. Simulations are run using $N = 10^5$, implying that an individual in the model maps to $\sim \$100,000$ (We verified that results do not depend on the choice of N, as long as N is large enough).

While in the neutral model a new species enters the system as a new individual, we further inform the model with the average size of a new cryptocurrency ($\sim$ \$1.5 million), corresponding to $m = 15$ individuals in the model when $N = 10^5$ as in our case. To consider the fact that new cryptocurrencies do not enter the market with exactly the same size, in our simulations, when a mutation occurs, the new species enters with a number m of individuals randomly extracted from the interval $[10, 20]$.

The exponent $\alpha = 1.5$ exhibited by the data and the simulations(see Fig. 5A, main text) are equilibrium properties of the neutral model, and hence obtained under a broad range of conditions (e.g., initial condition, time of start of measure and aggregation window) and robust to changes in the value of μ [1], Fig. S1). Fig. 5B and C in the main text are obtained starting from generation 104 and aggregating over 52 generations (i.e. performing the analysis over the single population obtained by aggregating the $N * 52$ individuals [2,3]). Fig. S2 shows the turnover profile (A) and average life time of a rank (B) when the measure is performed over 52 generations starting from different generations g_1 corresponding to the first year (measures start at generation $g_1 = 1$), second year (measures start at generation $g_1 = 53$), etc. It is clear that, with the exception of a high rank mobility characterizing the very first generations, the choice of g_1 has little effect on the curves produced by the model. Fig. 5D in the main text is measured from generation 1 up to generation 210, corresponding to 4 years. Each point of the simulation curve corresponds to the instantaneous market share of the dominating cryptocurrency at that generation.

3 Different technologies, same distribution

In order to check whether technical differences leave any detectable fingerprint at the level of statistical distributions, we look at cryptocurrencies adopting one of the two main blockchain algorithms for reaching consensus on what block represents recent transactions across the network: Proof-of-work (PoW) or the Proof-of-stake (PoS) consensus algorithms.

The PoW scheme was introduced as part of Bitcoin in 2009 [4]. To generate new blocks, participating users work with computational and electrical resources in order to complete “proof-of-works”, pieces of

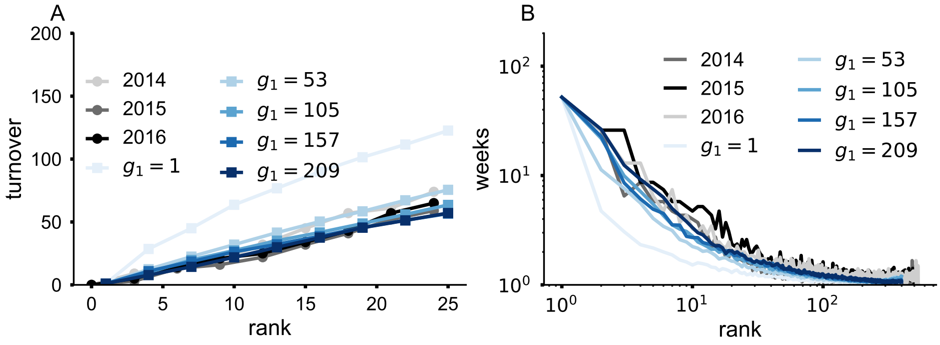

Figure S2: **Neutral model ranks dynamics.** **(A)** Turnover profile computed considering 52 for the cryptocurrencies data (gray lines, dots) and for numerical simulations (blue lines). **(B)** The Average life time a cryptocurrency/species stays in a given rank computed considering 52 generations for the cryptocurrencies data (gray lines, dots) and for numerical simulations (blue lines). Simulation parameters are $\mu = 7/N$, $N = 10^5$ and 1 species in the initial state.

data that are difficult to produce but easy to verify. Block generation (also called "mining") is rewarded with coins. To limit the rate at which new blocks are generated, every 2016 blocks the difficulty of the computational tasks changes [5].

While the PoW mechanism is relatively simple, there are concerns regarding its security and sustainability. First, severe implications could arise from the dominance of mining pools controlling more than 50% of the computational resources and who could in principle manipulate the blockchain transactions. This scenario is far from being unrealistic: in 2014, one mining pool (Ghash.io) [6] controlled 42% of the Bitcoin mining power. Also, the energy consumption of PoW based blockchain technologies has raised environmental concerns: it is estimated that Bitcoin consumes about 12.76 TWh per year [7].

The PoS scheme was introduced as an alternative to PoW. In this system, mining power is not attributed based on computational resources but on the proportion of coins held. Hence, the richer users are more likely to generate the next block. Miners are rewarded with the transactions fees. While proof-of-work relies heavily on energy, proof-of-stake doesn't suffer from this issue. However, consensus is not guaranteed since miners sole interest is to increase their profit. Through the years both protocols have been altered to fix certain issues and continue to be improved.

Figure S3 shows that the market shares of the two groups of cryptocurrencies follow the same behavior. The figure is generated using data collected from [8] and [9].

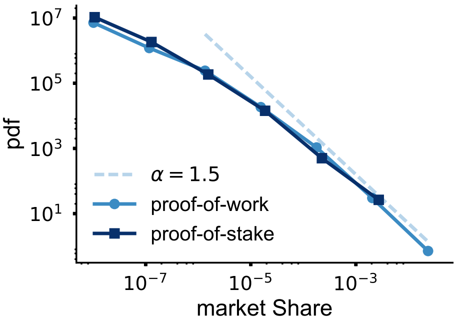

Figure S3: **Distribution of market share.** Distribution of the market share for proof-of-work cryptocurrencies (light blue filled line) and distribution of market share of (proof-of-stake or hybrid) cryptocurrencies (dark blue filled line). The dashed line is power law curve with exponent $\alpha = 1.5$.

4 Market share and frequency-rank distributions for individual years

The power-law fit for the distribution of market share (Table S2) and the frequency-rank distribution (Table S3) are consistent with the theoretical predictions of the neutral model [10] also for individual years. Fits coefficient for the distribution of market share are computed using the methodology described in [11] (errors are obtained by bootstrapping 1000 times). Fit coefficients with errors for frequency-rank distributions are computed with the least-square method.

Table S2: **Power-law fit coefficients of the market share distributions.**

**Year**	α
2013	1.37 ± 0.04
2014	1.54 ± 0.09
2015	1.62 ± 0.12
2016	1.59 ± 0.13
2017	1.60 ± 0.21
all years	1.58 ± 0.12

Table S3: **Power-law fit coefficients of the frequency-rank distributions.**

**Year**	β
2013	-1.98 ± 0.20
2014	-2.00 ± 0.13
2015	-1.83 ± 0.08
2016	-1.88 ± 0.08
2017	-1.86 ± 0.16
all years	-1.93 ± 0.23

References

[1] James P O'Dwyer and Anne Kandler. Inferring processes of cultural transmission: the critical role of rare variants in distinguishing neutrality from novelty biases. *arXiv preprint arXiv:1702.08506*, 2017.

[2] Matthew W Hahn and R Alexander Bentley. Drift as a mechanism for cultural change: an example from baby names. *Proceedings of the Royal Society of London B: Biological Sciences*, 270(Suppl 1):S120–S123, 2003.

[3] R Alexander Bentley, Matthew W Hahn, and Stephen J Shennan. Random drift and culture change. *Proceedings of the Royal Society of London B: Biological Sciences*, 271(1547):1443–1450, 2004.

[4] Satoshi Nakamoto. Bitcoin: A peer-to-peer electronic cash system. 2008.

[5] Pedro Franco. *Understanding Bitcoin: Cryptography, engineering and economics.* John Wiley & Sons, 2014.

[6] Bitcoin miners ditch ghash.io pool over fears of 51% attack. http://www.coindesk.com/bitcoin-miners-ditch-ghash-io-pool-51-attack/, 2014. Accessed: 5 June 2017.

[7] Bitcoin energy consumption index. http://digiconomist.net/bitcoin-energy-consumption, 2017. Accessed: 15 May 2017.

[8] Map of coins. https://www.mapofcoins.com, 2013. Accessed: 5 June 2017.

[9] coinmarketcap.com. http:https://coinmarketcap.com/, 2013. Accessed: 5 June 2017.

[10] Lada A Adamic and Bernardo A Huberman. Zipf's law and the internet. *Glottometrics*, 3(1):143–150, 2002.

[11] Aaron Clauset, Cosma Rohilla Shalizi, and Mark EJ Newman. Power-law distributions in empirical data. *SIAM review*, 51(4):661–703, 2009.
